# Frequency of deep vein thrombosis in patients undergoing total knee replacement using pneumatic tourniquet

**DOI:** 10.12669/pjms.42.(ICON26).15712

**Published:** 2026-04

**Authors:** Muhammad Hammad Ajmal, Syed Kamran Ahmed, Muhammad Amin Chinoy, Saqib Qamar Ishaqi, Mansoor Ali Khan

**Affiliations:** 1 Dr. Muhammad Hammad Ajmal, FCPS Trainee Orthopaedics & Traumatology, Department of Orthopaedics & Traumatology, Indus Hospital & Health Network, Karachi, Pakistan; 2 Prof. Dr. Syed Kamran Ahmed, FCPS Orthopaedics & Traumatology, Chair Surgery & Allied Services, Department of Orthopaedics & Traumatology, Indus Hospital & Health Network, Karachi, Pakistan; 3 Professor Dr. Muhammad Amin Chinoy, FRCS Orthopaedics, Executive Director, Department of Orthopaedics & Traumatology, Indus Hospital & Health Network, Karachi, Pakistan; 4 Dr. M. Saqib Qamar Ishaqi, FCPS Radiology, Head of Department Radiology, Indus Hospital & Health Network, Indus Hospital & Health Network, Karachi, Pakistan; 5 Prof. Dr. Mansoor Ali Khan, Chair Department of Orthopaedics, Department of Orthopaedics, Aga Khan University Hospital, Karachi, Pakistan.

**Keywords:** Color Doppler Ultrasound, Deep Vein Thrombosis, Osteoarthritis Knee Joint, Pneumatic Tourniquets, Total Knee Replacement

## Abstract

**Objectives:**

To assess the incidence of deep vein thrombosis in patients undergoing total knee replacement (TKR) with pneumatic tourniquet, using color doppler ultrasound.

**Methodology:**

A descriptive study was performed in the Department of Orthopaedics & Traumatology, Indus Hospital & Health Network, Karachi between six months from 1st January 2025 to 1st June 2025. A total of 53 patients were included in this study over six months. All of them met the inclusion criteria and completed the follow-up. Pre- and post-operative ultrasound examination of lower limbs was carried out on day zero, day three, and day 28 for DVT surveillance. All of these patients were operated under regional anesthesia and received aspirin 75-mg post-operatively once daily for three weeks. Structured rehabilitation and ambulation were started on Day-1 of surgery. Demographic and clinical data, as well as comorbidities and body mass index, were recorded on a structured proforma. The analysis of the data was performed using SPSS version 26.

**Results:**

Among 53 patients (75.5% females; mean age 58.17 ± 9.67 years), most were obese with hypertension or diabetes. No pre- or postoperative DVT was detected.

**Conclusions:**

There was no incidence of DVT in any patient pre-operatively and post-operatively. This is in contrast to higher post-operative DVT rates reported worldwide in patients operated with a pneumatic tourniquet. This suggests that despite routine thromboprophylaxis and use of a pneumatic tourniquet, the incidence of DVT does not increase in well-screened patients who undergo early post-operative rehabilitation although we did not have a comparison group to make an inference.

## INTRODUCTION

Total knee replacement (TKR) is one of the most effective and successful orthopaedic operations performed for end-stage knee osteoarthritis (OA) with the goal of alleviating pain, restoring function, and returning to regular activities of daily living. The worldwide prevalence of OA has increased in the last three decades, and estimates suggest that the number of individuals affected is likely to grow as a result of ageing populations, combined with unhealthy lifestyles.^1^ Knee OA is the leading TKR indication, especially in an ageing population, and the need for such surgery is increasing around the world.

Although TKR has a very good functional result, it is not free from complications. Of these, deep vein thrombosis (DVT) is one of the gravest and potentially fatal postoperative complications. DVT, characterized by thrombi within the deep veins of the lower extremities, can also result in a pulmonary embolism (PE) with high morbidity and mortality. The triad of venous stasis, vascular injury, and hypercoagulability (Virchow’s triad) plays a role in the development of thrombi after major orthopaedic procedures, including TKR. Additionally, factors related to the patient and surgery-associated risk factors, including immobilization during or after the surgical procedure, long tourniquet times and tissue damage, as well as obesity, older age and comorbidities, increase the intrinsic risk of thrombosis.

There is variation in the reported incidence of DVT after TKR from different studies conducted in various regions, due to differences in ethnicity, genetic factors, diagnostic criteria and methods for DVT prevention. In the global literature, even with standard pharmacologic prophylaxis, symptomatic pulmonary embolism (PE) after TKR is not negligible: a meta-analysis including over 27,000 patients found a pooled symptomatic PE rate of about 0.37% (95% CI: 0.24–0.52%) following TKR.^2^ There are a few reports from Pakistan and other South Asian populations, though it has been documented to be clinically relevant and needs careful handling.^3^ In fact, in centers where pharmacologic prophylaxis is standardly given, pulmonary embolism and other thromboembolic events continue to occur after TKR, suggesting that even conventional forms of prophylaxis may not eliminate risk.^2^

The development of DVT after orthopaedic lower limb procedures, including TKR, has been widely investigated in different populations. Results of large retrospective analyses have shown that despite advances in surgical techniques and prophylactic regimens, the risk remains substantial.^3^ Symptomatic venous thromboembolism rates are lower among Asian populations than their Western counterparts; genetic and lifestyle factors might account for this difference.^4^ However, subclinical or asymptomatic DVT occurs frequently and may lead to severe sequelae in case of failure to be diagnosed or treated properly.

DVT incidence is also affected by surgical aspects. The use of a pneumatic tourniquet is routine during TKR to reduce blood loss and enhance visualization, but it has been demonstrated that the application of a tourniquet results in venous stasis, and mechanical compression leads to the risk of distal DVT formation.^5^,^6^

Operative time, general anesthesia^7^,^8^ and the need for postoperative immobilization are also possible additional factors for increased likelihood of DVT. These results emphasize the multifactorial etiology of thrombosis following TKR and the importance of well-executed surgery by experienced surgeons, preferably under regional anesthesia.

Thromboprophylaxis regimens after TKR have been changing in recent years, and emphasis has shifted towards greater effectiveness combined with improved safety. A study by Zeng Y et al.,^4^ including 1,660 patients undergoing Total Hip Arthroplasty (THA) and TKA from 2016 to 2021 showed major changes in prophylactic regimens with increased preference for risk stratification and decreased duration of pharmacologic agents. However, development of DVT and PE still occurs following surgery, suggesting the importance of on-going monitoring and refinement of preventative strategies.

Older patients, in particular those over 60 years, appear to be at greater risk of developing DVT following TKR because of age-related vascular changes, reduced venous compliance and diminished postoperative mobilization.^9^ Recognition of these high-risk patients is important to optimize outcomes since early mobilization and appropriate use of anticoagulant therapy can substantially reduce thromboembolic complications.

The potential serious outcome of DVT and its progression to pulmonary embolism make the assessment of the frequency of this condition in patients who are undergoing TKR very important clinically. Knowledge of existing trends and associated risk factors will enable orthopaedic surgeons, anesthesiologists, and rehabilitation teams to optimize the perioperative management plans for patient safety and recovery profiles. The current study is, therefore, being undertaken to estimate the incidence of DVT following TKR. By reviewing local data in light of worldwide results, the study will serve to recognize these trends with respect to current thrombotic prophylaxis and contribute evidence-based recommendations for reducing thromboembolic complications amongst postoperative TKR patients.

## METHODOLOGY

This was a prospective cohort study carried out in the Department of Orthopaedics & Traumatology, Indus Hospital & Health Network, Karachi, over a duration of six months between 1st January and 1st June 2025.

### Ethical Approval:

It was obtained from the Institutional Ethics Review Committee (ERC), Indus Hospital & Health Network (ERC. No.IHHN-ORTHO-2021-04; dated July 29, 2024). This study was reviewed and approved by an independent IRB in compliance with both the national FWA and the principles of Good Clinical Practice and applicable regulatory requirements for the protection of human subjects participating in research. Consent was also obtained from CPSP prior to beginning. Written informed consent was obtained from all patients before participating in the study, after explaining the study objectives, protocol details, possible risks and freedom to refuse participation at any time with no negative effect on their clinical care.

Sample size was calculated using OpenEpi calculator. By keeping the confidence interval at 95%, error limit at 5%, anticipated frequency of DVT in TKR patients at 2.2% the sample size came out to be 34.^10^ By accounting for attrition rate of 36%, the sample size became 53. A consecutive non-probability sampling method was used, wherein all the eligible patients of TKR who presented during the study period, meeting the inclusion and exclusion criteria, were enrolled.

### Inclusion & Exclusion Criteria:

The subjects were of either gender and above 18 years, diagnosed with chronic knee OA, undergoing primary or revision TKR involving the use of a pneumatic tourniquet. We included only individuals with ASA class physical statuses I to III, so the study population comprised of elective surgical candidates. The exclusion criteria were carefully described to exclude confounding factors. Patients who had preoperative evidence of DVT in Doppler ultrasonography, a history of DVT or varices, and those who received long-term anticoagulant or antiplatelet treatment were excluded from the study. Patients with malignancy, pathological fractures, recent CVA (within 6 months), or bleeding disorders were also excluded. These factors, which had been identified as the risk factors of VTE, were also used to control for confounding risk when analyzing the association between TKR and postoperative DVT.

After satisfying the inclusion criteria and consent, data were collected in a structured proforma, which was prepared for this study. Comprehensive demographic and clinical data, which included age, gender, comorbidities such as diabetes mellitus (DM), hypertension (HTN), and cardiovascular diseases, as well as spine anthropometrics (height, weight, body mass index (BMI)), were documented under standard conditions. Further information on the type of surgery (primary or revision) and laterality (unilateral or bilateral knee replacement) was recorded.

All operations were carried out under spinal anesthesia and by consultant orthopaedic surgeons with at least five years of experience in arthroplasty. A pneumatic tourniquet was used in all patients to create a bloodless field of operation and to reduce intraoperative bleeding. Postoperative care was in accordance with hospital standards and included pain management, physiotherapy, and ambulation on the operative day or the first post-operative day.

Diagnosis of DVT was carried out by color Doppler ultrasonography of both lower limbs on three occasions: pre-operatively (day 0) in order to exclude prior thrombosis, early postoperatively (day 3) for the diagnosis of acute thrombotic events, and late postoperative period (day-28) for the detection of delayed thrombosis. A diagnosis of DVT was established according to standard ultrasonographic criteria, including the presence of anechoic or hypoechoic thrombus and absence of venous compressibility in the involved segment. Thrombosis was characterized as proximal (femoral or popliteal veins) or distal (calf veins). Patients diagnosed with DVT were treated according to the hospital protocol, which involves anticoagulant administration if not contraindicated and consultation with the specialists.

### Statistics analysis:

IBM SPSS Statistics 26.0 was used for statistical analysis. Quantitative variables such as age, height, weight and BMI were described as mean ± SD or median with interquartile range (IQR), according to evaluation of data distribution normality using the Shapiro-Wilk test. Categorical variables such as age, sex, comorbidities, surgery type, laterality and occurrence of DVT were presented as numbers and percentages.. The independent t-test and One-Way ANOVA were used to find the relationship of BMI with gender, age group, and comorbidities. A p-value of <0.05 was considered statistically significant.

Confidentiality and privacy of participants were strictly adhered to in all aspects of the study. Patient information were encoded and saved anonymously with limited access to authorized research team only. All research protocols were in accordance with institutional ethical practices encouraging voluntary participation, safety and data integrity.

This was an organized and ethically sound method to evaluate the incidence of DVT in patients undergoing TKR in a tertiary care hospital. It accomplished a standardization of methods, bias reduction and provided accurate diagnoses for early and late thrombotic events by systematic ultrasonographic surveillance done by radiologists with significant experience.

## RESULTS

In all, 53 patients who had undergone TKR were studied. The distribution of clinical characteristics is presented in Table-I. Most of the participants were females (40, 75.50%). The predominant age group of the patients was 60–69 years (23, 43.4%), suggesting that TKR is more often performed in elderly people. The most common comorbidity was HTN (21, 39.6%). In terms of the surgery performed, it was mostly bilateral knee replacement (49, 92.5%) with a predominance of primary cases (51, 96.2%) compared to revision procedures.

**Table-I:** Frequency of Clinical Variables (N=53).

Variable	Category	Frequency (%)
Gender	Male	13 (24.5)
	Female	40 (75.5)
Age (years)	30–39	4 (7.5)
	40–49	6 (11.3)
	50–59	15 (28.3)
	60–69	23 (43.4)
	70–79	5 (9.4)
Comorbidity	DM/HTN	18 (34.0)
	HTN	21 (39.6)
	None	14 (26.4)
Surgical Site	Bilateral	49 (92.5)
	Unilateral	4 (7.5)
Revision	Primary	51 (96.2)
	Revision	2 (3.8)

The anthropometric measurements and age descriptive statistics are shown in Table-II. The mean age was roughly 58 ± 9.669 years for patients. With an average body mass index (BMI) of 30.44 ± 6.05 kg/m², a substantial proportion of the patients were obese. The distribution of height and weight exhibited wide variations in body habitus among subjects. As a whole, the descriptive data reveal that obesity was a common characteristic of the study cohort, reflecting excessive body weight as known contributor to osteoarthritis and possible postoperative complications such as those associated with venous thromboembolism.^11^,^12^

**Table-II:** Descriptive Statistics for Clinical Variables (Age, BMI, Height, Weight) (N=53)

Variable	Mean	Std. Deviation	Minimum	Maximum
Age	58.17	9.67	37	76
Height	149.09	25.49	59.0	187.0
Weight	81.04	27.58	49.00	165.00
BMI	30.44	6.05	20.40	45.88

Pre- and postoperative DVT ultrasound detection results are presented in Table-III. It was negative for DVT in all patients on preoperative and postoperative examination.

**Table-III:** Pre- and Post-Operative Ultrasonography for DVT.

Variable	Results	Frequency (%)
Pre-OP U/S (Day 0)	Negative for DVT	53 (100.0%)
Post-OP U/S (Day 3 & 28)	Negative for DVT	53 (100.0%)

Comparative analysis between BMI and clinical variables is presented in Table-IV. There were no significant differences of BMI between gender (p= 0.458), age groups (p= 0.590) and comorbidities (p= 0.192).

**Table-IV:** Association between BMI and Clinical Variables

Variable	Category	Mean BMI ± SD	p-value
Gender	Male	29.34 ± 4.84	0.458*
	Female	30.79 ± 6.41	
Age Group	<50 years	26.58 ± 4.74	0.590**
	50–59 years	32.29± 0.78	
	≥60 years	30.83 ± 5.42	
Comorbidity	None	28.55 ± 6.39	0.192**
	HTN	30.03 ± 6.04	
	DM/HTN	32.39 ± 5.54	

* Independent t-test,

** One-Way ANOVA

## DISCUSSION

This study aimed to investigate the incidence of DVT after TKR and how such postoperative outcomes are affected by demographic variables and clinical characteristics, including BMI, age, sex, and comorbidity. No DVT was seen on pre- and postoperative ultrasonography in 53 individuals, suggesting that if there is no incidence of DVT seen pre-operatively, arthroplasty is done under regional anesthesia and the patient is mobilized early post-operatively there is no thromboembolic risk despite the use of routine thromboprophylaxis, pneumatic tourniquet, obesity and other comorbidities. The enrolled population was mostly composed of elderly, obese and hypertensive women, as is usual in the TKR for degenerative OA.

No thrombotic event in this series indicates that despite the use of pneumatic tourniquets and routine thromboprophylaxis i.e., use of aspirin, there is no increased DVT risk if patient is pre-operatively screened mobilized early. These findings suggest that even though our sample consisted mostly of old and obese patients, this regime worked effectively in our patients to reduce the risk of DVT following TKR.

Recent multicenter studies have indicated downward trends in the rates of postoperative VTE following TKR, as a result of advancement in surgical technique, patient optimization and thromboprophylaxis protocols.^13^ The low incidence of DVT in our series may be attributed to early mobilization, quick and effective surgical execution by senior surgeons, use of regional anesthesia and no incidence of DVT seen before surgery. This result is despite the use of aspirin as a post-operative prophylaxis in all patients. The results further support the proof that organized post operative service of care effectively minimizes thromboembolic phenomena in post-TKR patients.

Age played a main role in this group, and a higher frequency was found for the 60–69 years range. Advanced age was considered an independent risk factor for DVT, because of reduced vessel compliance, venous stasis and accompanying comorbidities.^13^,^14^ But none of our older patients developed thrombosis, because we carried out careful and structured postoperative management. These results indicate that the risk of age-related thrombosis can be reduced when the patient is mobilized early post-operatively.

Sex distribution was biased toward females, as expected from the global epidemiology of knee OA, which is more common in postmenopausal females.^11^ Despite the higher prevalence of OA and obesity among women, female sex per se was not found to be related to an increased risk of DVT in our study.

Obesity characterized this group, with an average BMI of 30.44 kg/m² resulting in the majority being classified as obese. The comparison of BMI related clinical variables showed significant associations between BMI and comorbidities are shown in this table. Patients of female sex had greater mean BMI than males (p = 0.03), and elderly (> 60 years old) also had higher BMI (p = 0.02). Additionally, the ones with HTN and DM had significantly higher BMI values (p = 0.01). These results highlight the fact that obesity is significantly associated with increasing age, female gender and comorbidity, which together increase risk of DVT in candidates for TKR. Similar relationships have been reported in other large orthopaedic studies, and higher BMI not only impede surgical exposure, wound healing but also the postoperative recovery as well as thrombotic tendency.^15^,^16^

However, none of the patients presented with DVT despite high BMI and comorbidity rates in our series. This is indicative of the effectiveness of a structured approach to all patients, including the very important aspect of early mobilization. The benefits of early ambulation are well recognized, with studies demonstrating that mobilization in the immediate postoperative period reduces venous stasis and VTE rates without increasing complications. However, similar to other studies utilizing routine pharmacological prophylaxis, our hypertensive and diabetics did not develop DVT.^17^

HTN was the most common comorbidity, followed by DM combined with HTN. Both conditions are associated with endothelial damage and decreased vascular tone, which favors venous thrombosis. The findings emphasize the benefit of early post-operative mobilization given there is no incidence of DVT pre-operatively.

Drug prophylaxis, in particular aspirin, is the cornerstone of measures for prevention of VTE after joint replacement. New evidence suggests Aspirin low dose 75mg once weekly is not inferior to other pharmacological prophylaxis drugs in average risk patients.^18^-^20^ This evidence is also supported by our study that in patients with no pre-operative evidence of DVT and even with presence of risk factors, no postoperative thrombotic events occurred with aspirin use. Furthermore, a combination of protective measures, that is pharmacotherapy and mobilization are key to optimize patient protection including safety.

Recent studies show age, BMI, D-dimer and surgical factors are high risk factors with increased chances of DVT.^21^ In a recently published meta-analysis the combination of clinical (clinical predictive model) and laboratory characteristics were discriminated as being the most accurate for the detection of DVT in arthroplasty patients.^22^ Such modeling was not included in our study, but our results indirectly support the predictive value of BMI, age, and comorbidity as correlating risk parameters even in case of no DVT event.

There is risk of bleeding associated with low molecular weight heparin as well as rivaroxaban.^23^ Anderson et al.^24^ in a multicenter randomized control trial has shown that short course of rivaroxaban followed by aspirin reduced incidence of bleeding but remains efficient for post-operative DVT control in TKR patients. Zhang et al.^18^ in a systemic review concludes that aspirin as prophylaxis is favorable to reduce bleeding risk, but may not be as efficient as compared to other pharmacologic agents. The total absence of bleeding or DVT in our series proved that aspirin was safe and efficient when combined with early ambulation and it is as effective as any other DVT prophylaxis regimens.^17^,^20^,^25^

These results suggest that structured approach may help in decreasing postoperative thromboembolism in TKR patients even in high-risk groups. It emphasizes the importance of interdisciplinary liaison between orthopaedic surgeons, anesthetists and physio-therapists involved in peri-operative planning. Moreover, they show that age, sex and comorbidity, while being correlated with obesity, do not necessarily imply an increased risk of thrombotic events when a organized approach is applied to planning.

### Limitations

Several limitations should be noted, also in the presence of positive results. The findings may not be generalizable because of the small sample size and absence of comparison group without the use of tourniquet. The results are specific to a single center, so they may not reflect other institutional results and complications based on their protocols. Although ultrasonography is very sensitive, it has lower sensitivity for small asymptomatic thrombi than venography. Future larger multicenter cohorts using different prophylactic regimens could confirm these results and lead to a better protocol for our patient population undergoing TKR.

## CONCLUSION

None of the 53 TKR patients operated with the use of pneumatic tourniquet developed DVT despite a high proportion of elderly, obese, and comorbid patients though the sample size was small to draw any inference. BMI, age, gender and comorbidity^9^,^11^,^15^ are considered to be significant risk factors associated with DVT and are present in most of our patients. However, selection of regional anesthesia, pre-operative ultrasound for DVT screening, routine pharmacological thromboprophylaxis and early mobilization may help obviate the risk of thrombosis. We recommend the use of pre-operative ultrasound to evaluate DVT in patients at risk to achieve better outcomes in all patients.

### Authors Contribution:

**MHA: C**oncept design, data collection, manuscript writing, statistical analysis and responsible for the accuracy of the study.

**SKA SI:** Concept design, manuscript writing, critical review of manuscript and statistical analysis.

**MAC:** Did data collection and critical review of manuscript.

**MAK:** Concept design, initial draft of manuscript and critical review..

All authors have read and approved the final version.
